# The role of neuroimaging in the diagnosis of cerebral toxoplasmosis: a systematic review

**DOI:** 10.1590/0100-3984.2025.0096

**Published:** 2026-06-29

**Authors:** Pedro Henrique Abreu Tiradentes, Luã Portela Carvalho, Mariana Dalaqua, Tomás de Andrade Lourenção Freddi, Lázaro Luís Faria do Amaral, Gustavo Kazuo Yamada, Simone Appenzeller, Juliana Avila Duarte, Fabiano Reis

**Affiliations:** 1 Department of Radiology and Oncology, Universidade Estadual de Campinas, Campinas, SP, Brazil; 2 Department of Radiology, Réseau Hospitalier Neuchâtelois, Neuchâtel, Switzerland; 3 Hospital do Coração, São Paulo, SP, Brazil; 4 Hospital Beneficência Portuguesa, São Paulo, SP, Brazil; 5 Department of Orthopedics and Rheumatology, Universidade Estadual de Campinas, Campinas, SP, Brazil; 6 Department of Radiology and Diagnostic Imaging, Hospital de Clínicas de Porto Alegre (HCPA), Porto Alegre, RS, Brazil

**Keywords:** Toxoplasma, Toxoplasmosis, cerebral/diagnosis, Toxoplasmosis/diagnostic imaging, Neuroimaging., Toxoplasma, Toxoplasmose cerebral/diagnóstico, Toxoplasmose/diagnóstico por imagem, Neuroimagem

## Abstract

This study presents a systematic review of the role of imaging in the diagnosis of central nervous system toxoplasmo-sis, in addition to a case series from a tertiary university hospital. The review was conducted in accordance with the Preferred Reporting Items for Systematic reviews and Meta-Analyses guidelines and registered in the International Prospective Register of Systematic Reviews (ID: CRD420251107718). Studies published after January 1, 2000 were retrieved from the PubMed, Embase, Scopus, and Latin-American and Caribbean Health Sciences Literature data-bases. Two authors, working independently, selected studies that met the eligibility criteria, which were organized with Rayyan software. Imaging findings were described using computed tomography (CT), conventional magnetic resonance imaging (MRI), advanced MRI sequences or nuclear imaging, focusing on single-photon-emission com-puted tomography, with and without thallium-201, and positron-emission tomography/CT. Extracted data focused on lesion topography, signal characteristics, enhancement, restricted diffusion, and when available, spectroscopy and perfusion MRI findings. To illustrate typical and atypical imaging features, a complementary case series was included, evaluating patients with confirmed cerebral toxoplasmosis. The review demonstrates that CT and MRI remain es-sential for diagnosis and follow-up, whereas advanced MRI sequences provide additional value in differentiating toxo-plasmosis from other opportunistic infections and neoplastic processes.

## Abstract (pt)

Este estudo apresenta uma revisão sistemática do papel da imagem no diagnóstico da toxoplasmose do sistema ner-voso central, além de uma série de casos de um hospital universitário terciário. A revisão foi conduzida de acordo com as diretrizes *Preferred Reporting Items for Systematic reviews and Meta-Analyses* e registrada prospectivamente no *International Prospective Register of Systematic Reviews* (ID: CRD420251107718). Estudos publicados após 1º de janeiro de 2000 foram recuperados do PubMed, Embase, Scopus e Literatura Latino-Americana e do Caribe em Ciências da Saúde. Dois autores independentes selecionaram estudos que atendiam aos critérios de elegibilidade, os quais foram organizados usando o Rayyan.ai. Os achados de imagem foram descritos usando tomografia com-putadorizada (TC), ressonância magnética (RM) convencional, sequências avançadas de RM ou medicina nuclear, com foco em tomografia computadorizada por emissão de fóton único, com e sem cloreto de tálio-201, e tomografia por emissão de pósitrons/TC. Os dados extraídos focaram na topografia das lesões, características de sinal, realce, re-strição à difusão e, quando disponíveis, achados de espectroscopia e perfusão. Para ilustrar características de ima-gem típicas e atípicas, foi incluída uma série de casos complementar, referente a pacientes com neurotoxoplasmose confirmada. A revisão demonstra que a TC e a RM permanecem essenciais para o diagnóstico e acompanhamento, enquanto sequências avançadas de RM fornecem valor adicional na diferenciação da toxoplasmose de outras in-fecções oportunistas e neoplasias.

## INTRODUCTION

**Figure 1:**
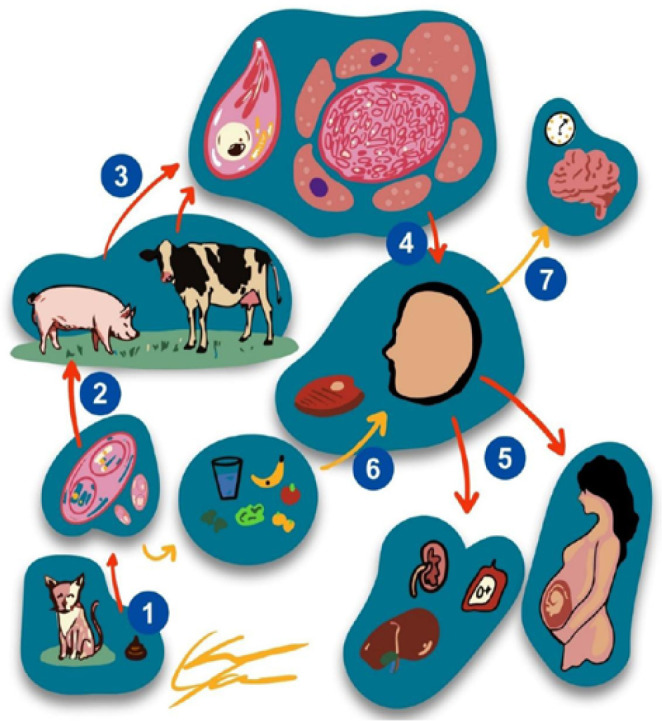
Illustration representing the life cycle of *Toxoplasma gon-dii*. 1, Shed as oocysts in cat feces (definitive hosts); 2, Ingested by livestock (intermediate hosts); 3, Develops into tachyzoites and then to tissue cysts containing bradyzoites; 4, Humans ingest cysts via undercooked meat; 5, Tachyzoites and bradyzoites can be transmit-ted via blood transfusion and organ transplantation; 6, Ingested by humans (intermediate hosts), oocysts develop into tachyzoites and then into tissue cysts containing bradyzoites; 7, Bradyzoites remain quiescent, even in the CNS, although they can reactivate after long periods in an immunocompromised condition. Original art by Gus-tavo Kazuo Yamada, 2024.

*Toxoplasma gondii* is a protozoan of the phylum Api-complexa whose definitive hosts are cats. However, it can infect many intermediate hosts, including all mammals and birds. Humans are typically infected by ingesting oocysts in food, acting as definitive hosts, or consuming undercooked meat containing viable cysts, acting as inter-mediate hosts. Transmission can also occur through blood transfusions and organ transplants, as well as through vertical transmission, leading to cases of congenital toxo-plasmosis^**(^1^,^2^)**^. Figure 1 illustrates the life cycle of *T. gondii*. It is, therefore, a highly prevalent, ubiquitous infection, with approximately 25-30% of the global population cur-rently infected by the parasite, being most prevalent in Latin American and African nations^(^3^-^5^)^. Despite its high prevalence, most acquired forms of toxoplasmosis in immunocompetent individuals are mild^**(^6^)**^. However, latent stages of the protozoan can remain dormant and later reactivate in immunosuppressed and congenitally infected individuals, primarily targeting the central nervous system (CNS). In addition, toxoplasmosis is the leading cause of cerebral mass lesions in immunosuppressed patients with HIV/AIDS who are not receiving appropriate prophylaxis. According to the literature, most cases of cerebral toxo-plasmosis in HIV-infected patients are associated with low CD4+ counts, especially below 100 cells/µL^**(^7^-^9^)**^. Cerebral toxoplasmosis also occurs in other forms of immunosuppression, most notably in individuals using immunosuppressive agents for the treatment of autoim-mune diseases, with a wide variety of cases being reported in the literature. Durieux et al.^**(^10^)**^ described a multicenter analysis and literature review on this subject, concluding that the most common drugs associated with cerebral toxoplasmosis were corticosteroids, antimetabolites, and anti-tumor necrosis factor alpha. Furthermore, reports in the literature have showcased cerebral toxoplasmosis as a possibility in patients treated with biologic agents such as the monoclonal antibodies rituximab, adalimumab, and infliximab^**(^11^,^12^)**^. It is important to differentiate between the manifestations of cerebral toxoplasmosis and the primary neuropsychiatric manifestations seen in systemic lupus erythematosus^**(^13^)**^.

Transplant recipients are also affected by cerebral toxoplasmosis, including those undergoing solid organ transplantation, especially kidney, liver, and heart trans-plant recipients, and those undergoing hematopoietic stem cell transplantation (HSCT). A multicenter analysis of transplant recipients in Europe showed that the preva-lence of cerebral toxoplasmosis was highest among HSCT recipients^**(^14^)**^, among whom it has been found to be the most common CNS infection^**(^14^,^15^)**^. In patients with cancer, toxoplasmosis is also a possibility, albeit a relatively uncom-mon opportunistic disease. Among such patients, cerebral toxoplasmosis is most common in those with hematological malignancies, especially Hodgkin lymphoma^**(^16^)**^.

In patients with cerebral toxoplasmosis, the most common symptoms are headaches, fever, focal deficits, hemiparesis, ataxia, cranial nerve palsies, changes in con-sciousness, and seizures. More rarely, chorea, ballismus, and rigidity can be observed^**(^6^,^7^)**^.

A definitive diagnosis of cerebral toxoplasmosis can be made only through histopathological analysis of brain biopsies, the gold-standard diagnostic modality, or mo-lecular assays to identify *T. gondii* DNA in cerebrospinal fluid (CSF). However, these procedures have considerable limitations: biopsies are invasive and depend on the loca-tion of the lesion and the clinical condition of the patient; and access to molecular assays is limited (especially in lowand middle-income countries). Although polymerase chain reaction of CSF has high (96-100%) specificity for diagnosing cerebral toxoplasmosis, it is significantly less sensitive and a negative result therefore cannot rule out the disease^**(^6^,^7^,^17^)**^. In fact, evaluation of the CSF is irrelevant in diagnosing the disease, given that toxoplas-mosis rarely involves the meninges, and CSF changes are usually nonspecific. Similarly, serological diagnosis is not particularly elucidative, because cerebral toxoplas-mosis is related to reactivation of latent infection and immunoglobulin M (IgM) titers are therefore negative. In addition, the presence of *T. gondii* IgG antibodies is inconclusive, because high IgG titers do not necessarily indicate the presence of *T. gondii*, and the absence of such antibodies is also insufficient to rule out cerebral toxoplasmosis^**(^6^-^8^)**^. Therefore, neuroimaging plays a piv-otal role in the diagnosis and emerges as an important resource in overcoming these limitations. It establishes a presumptive diagnosis of the disease by identifying one or more brain lesions that are consistent with the disease and improve after 10-14 days of specific treatment^**(^7^,^8^,^18^)**^.

The aim of this study was to assess the importance of neuroimaging methods in the correct diagnosis of cerebral toxoplasmosis. To that end, we conducted a systematic review of the literature and a retrospective analysis of confirmed cases from a tertiary university hospital.

## METHODS

### Protocol and registration

This systematic review was conducted in accordance with the Preferred Reporting Items for Systematic reviews and Meta-Analyses guidelines. The review protocol was registered in the International Prospective Register of Systematic Reviews (ID: CRD420251107718).

### Search strategy and study selection

Studies were selected from the following databases: Embase; Latin-American and Caribbean Health Sci-ences Literature; PubMed, and Scopus. Only studies published in English, Spanish, or Portuguese were in-cluded. The search terms included the following: cere-bral toxoplasmosis, neurotoxoplasmosis; central nervous system toxoplasmosis; magnetic resonance imaging; MRI; diffusion-weighted imaging; DWI; magnetic resonance spectroscopy; perfusion imaging; computed tomography; CT; CT scan; diagnosis; diagnostic imaging; diagnostic accuracy; sensitivity; and specificity.

The AI-powered software Rayyan (Qatar Computing Research Institute, Doha, Qatar) was used in order to organize the selected studies. Two researchers, each with at least 5 years of experience in the field, conducted the database searches and study selection. The two research-ers, working independently, assessed the eligibility of the articles found, with discrepancies being resolved through discussion. The initial screening was based on the titles and abstracts of the paper, and the full texts were then analyzed to determine whether they include analyses if imaging methods, for inclusion or exclusion.

### Eligibility criteria

The review included studies involving human pa-tients of any age, gender, or immunological status, with a confirmed or suspected diagnosis of cerebral toxoplas-mosis, based on clinical, laboratory, or radiological crite-ria. Eligible studies included those employing magnetic resonance imaging (MRI) or computed tomography (CT) of the brain or spine, using conventional sequences-T1-weighted imaging (T1WI), T2-weighted imaging (T2WI), fluid-attenuated inversion recovery (FLAIR) sequences, or contrast-enhanced sequences-or advanced MRI tech-niques-diffusion-weighted imaging (DWI), spectroscopy, or perfusion MRI). Studies featuring imaging findings obtained from other nuclear imaging modalities, such as single-photon-emission computed tomography (SPECT), with or without thallium-201, and positron-emission tomography/CT (PET/CT), were also included. We in-cluded only observational studies (cohort, case-control, or cross-sectional studies) and case series with five or more patients. Therefore, case reports with fewer than five patients, reviews, editorials, animal studies, and *in vitro* studies were excluded, as were studies involving patients with CNS coinfections (e.g., cryptococcosis or tuberculosis), without separate data for toxoplasmosis, and studies with insufficient neuroimaging data.

### Data extraction

In this retrospective study, we reviewed CT, MRI, PET/CT and SPECT scans of cases with a confirmed or suspected diagnosis of toxoplasmosis. In addition to data from the literature, illustrative CT and MRI scans of patients with confirmed cerebral toxoplasmosis from a tertiary university hospital were included in order to exemplify typical and atypical findings. Structural images and advanced sequences were obtained in 1.5-T and 3.0-T MRI scanners (Achieva; Philips Medical Systems, Best, the Netherlands). The following characteristics were analyzed by an experienced neuroradiologist (with over 20 years of experience): topography; diffusion-weighted aspects; T1WI and T2WI signal intensity; enhancement pattern; spectroscopy findings (if available); and perfusion MRI findings (if available). No authors were contacted for missing information, and no additional data beyond those published were requested.

### Data synthesis

The findings were summarized in narrative format and in structured tables. The focus was on information related to imaging modalities, imaging findings, and di-agnostic accuracy.

## RESULTS

**Figure 2:**
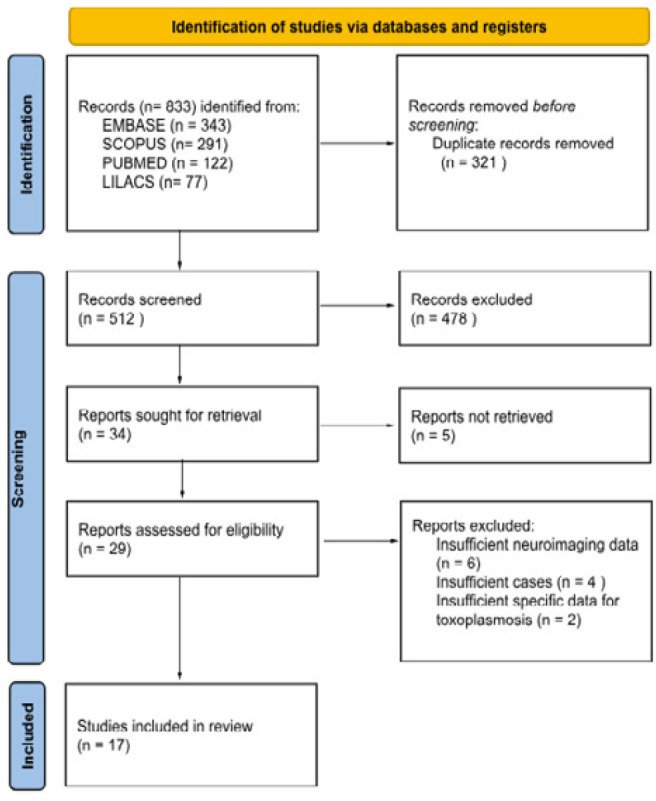
Flow chart of the article selection process. LILACS, *Lit-eratura Latinoamericana y del Caribe en Ciencias de la Salud* (Latin-American and Caribbean Health Sciences Literature).

Via our search strategy, 833 studies were selected. From those, 321 duplicates were removed. During the initial screening, titles and abstracts were reviewed, re-sulting in the exclusion of 478 articles. In addition, five studies were inaccessible. Therefore, 29 studies were included in the full-text analysis. Finally, after the full manuscript review, 17 studies were found to meet the eligibility criteria and were selected for inclusion in the systematic review. Figure 2 shows the article selection process. The details of the selected studies are shown in Table 1. The results of the review are summarized in Table 2 and Table 3.

**Table 1:** Characteristics of the studies evaluated.

Reference	N	Mean age (years)	Male (%)	Imaging method(s)
Dibble et al.**^(^19^)^**	5	46	60	Dynamic susceptibility contrast MRI
Batra et al.**^(^20^)^**	8	35	100	MRI, DWI, Spectroscopy, and perfusion MRI
Li et al.**^(^21^)^**	7	35.8	86	MRI, DWI, SWI, 3D contrast-enhanced T1WI
Hakko et al.**^(^15^)^**	5	47.3	80	T1WI and FLAIR MRI
Vidal et al.**^(^22^)^**	55	36	60	CT and MRI
Shyam babu et al.**^(^23^)^**	21	38.3	95	CT, MRI, and SPECT
Young et al.**^(^24^)^**	12	42	75	CT, MRI, and SPECT
Bhagavati & Choi**^(^25^)^**	11	42.4	57	CT and MRI
Romero et al.**^(^26^)^**	18	NI	67	MRI
Lau et al.**^(^27^)^**	38	38.8	55	MRI
Coleman et al.**^(^28^)^**	24	36.5	67	MRI
de Oliveira et al.**^(^29^)^**	56	39.1	52	CT and MRI
Benson et al.**^(^30^)^**	15	44	87	CT and MRI
Pradhan et al.**^(^31^)^**	15	48	80	MRI
Schroeder et al.**^(^32^)^**	22	NI	NI	MRI and DWI
Westwood et al.**^(^33^)^**	6	33.5	83	18F-FDG PET/CT and MR spectroscopy
Achappa et al.**^(^34^)^**	33	37.3	88	CT and MRI

N, number of participants; 3D, three-dimensional; NI, not informed.

**Table 2:** Summary of imaging findings from the studies evaluated. Imaging finding(s) N of studies

Imaging finding(s)	N of studies
Enhancement pattern	
Ring enhancement	9
Other enhancement patterns (nodular, discoid, meningeal)	4
Eccentric target sign	2
Associated findings	
Perilesional edema	5
Elemorrhagic transformation/foci	4
Mass effect/midline shift	2
Ventricular dilation/hydrocephalus	1
Signal characteristics (MRI)	
T2/FLAIR-hyperintense lesions	4
Diffusion abnormalities (restricted or increased)	4
Tl-hypointense lesions	3
Tl-hyperintense lesions (suggesting hemorrhage)	2
Susceptibility signal foci (SWI)	2
CT findings	
Hypodense lesions	2
Hyperdense lesions	1
Nuclear medicine & spectroscopy	
Low perfusion or reduced metabolic activity	4
Abnormal spectroscopy peaks (lipid, choline, lactate)	2

**Table 3:** Summary of lesion characteristics.

Finding	N of Studies
Multiple lesions	12
Solitary lesions	1
Lesion location	
Basal ganglia	6
Frontal lobe	5
Grey-white matter junction/ subcortical white matter	4
Parietal lobe	3
Cerebellum	3
Thalamus	2
Pons	1

The mean age of the patients involved was 39.6 years (range, 33.5-48.0 years). In most of the study samples, there was a predominance of males, with proportions as high as 80-90% in some samples. The majority of patients were immunocompromised, primarily due to advanced HIV/AIDS, and some samples included patients who had undergone transplantation or had other immunosuppres-sive conditions.

The most common CT finding was that of hypodense lesions with surrounding vasogenic edema and mass effect, typically showing ring or nodular contrast enhancement. The use of MRI revealed hypointense lesions on T1WI and hyperintense signals on T2/FLAIR sequences, with characteristic peripheral ring enhancement. The eccentric target sign was cited in only two studies, with no mention of the concentric target sign^**(^22^,^24^)**^. The DWI findings were varied, with some studies reporting restricted dif-fusion and others suggesting mixed or absent restriction; susceptibility-weighted imaging (SWI) typically showed complete, smooth, low-signal-intensity rims; perfusion MRI revealed low relative cerebral blood volume; and MR spectroscopy commonly demonstrated an elevated lipid peak with reduced choline and N-acetylaspartate.

Nuclear medicine studies were important in differentiating toxoplasmosis from primary CNS lymphoma. In many cases, technetium-99m (^99m^Tc) SPECT suggested inflammatory lesions; thallium-201 (201Tl) SPECT showed lower uptake in toxoplasmosis-related lesions than in lymphoma-related lesions; and ^18^F-fluorodeox-yglucose (^18^F-FDG) PET/CT consistently demonstrated low or nonexistent intralesional metabolic activity, thus differentiating it from the elevated activity present in lymphoma lesions.

In the majority of studies, multiple lesions were described as the most common pattern in cerebral toxo-plasmosis. Only one study cited solitary lesions as being the more common presentation, and there were four studies in which the pattern of lesion distribution was not reported^**(^31^)**^. Seven studies provided information on the total number of lesions; the mean number of lesions per patient was 5.47. The most common lesion locations were the basal ganglia and frontal lobe, whereas the least common sites were the pons and the deep grey matter.

## DISCUSSION

Images were collected to demonstrate the most com-mon patterns and some differential diagnoses.

### CT

**Figure 3:**
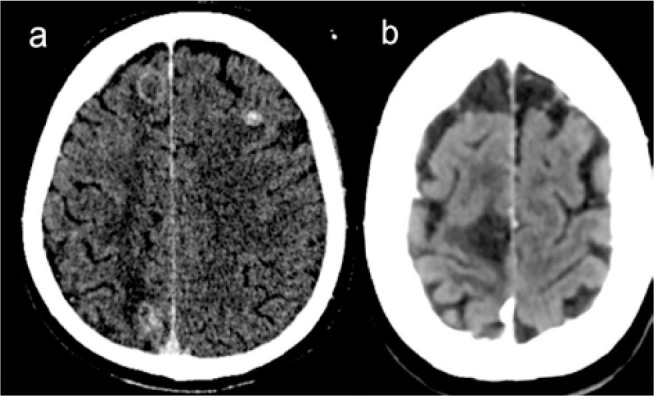
Contrast-enhanced computed tomography. **A:** Hypodense lesions with ring and nodular enhancement, together with perile-sional edema (a typical pattern of toxoplasmosis). **B:** Hypodense lesions without significant contrast enhancement and with a mild expansive effect (an atypical pattern of toxoplasmosis).

Despite being less sensitive than MRI, especially for smaller lesions, CT can also be used for the initial diagnosis of cerebral toxoplasmosis. The typical pattern observed on CT (in approximately 80% of cases) is one of hypodense lesions with perilesional vasogenic edema, with ring or nodular enhancement on contrast-enhanced images. Atypical patterns on CT (in approximately 20% of cases) include hypodense lesions without enhancement and with mass effect and diffuse cerebral edema without visible focal lesions. The most common lesion locations on CT are the basal ganglia and corticomedullary junction (in approximately 85% of cases), with multiple lesions^**(^35^,^36^)**^. Figure 3 shows lesions at the corticomedullary junction.

### MRI

The gold-standard imaging modality for evaluating patients with suspected cerebral toxoplasmosis is MRI. Studies^**(^6^,^8^,^35^)**^ have proven that MRI is more sensitive than is CT, because it can accurately diagnose lesions that would be otherwise misinterpreted. In addition, MRI has a higher histopathological correlation, representing a reliable option with fewer false-negative results. On MRI, findings of expansile mass lesions, with peripheral (ring) enhancement, often with an eccentric mural nodule, in patients with immunosuppression indicate cerebral toxoplasmosis as the most likely diagnosis, with differen-tial hypotheses being considered only when there is no response within the first 14 days of the specific treatment for toxoplasmosis.

**Figure 4:**
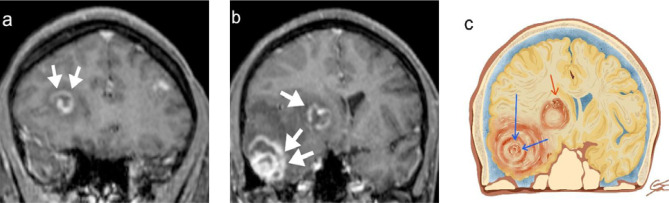
MRI of a 37-year-old male AIDS patient with left hemiparesis. Contrast-enhanced axial and coronal T1WI (**A** and **B**, respectively) showing two fo-cal ring-enhancing lesions with the eccentric target sign. One is located in the right lenticular nucleus (single arrow), and the other is in the right periventricu-lar frontal region (double arrows). Panel **C** shows an original illustration of the lesions in the right lenticular nucleus (red arrow) and in the right temporal lobe (blue arrows), with a concentric pattern of enhancement.Original art by Gustavo Kazuo Yamada.

**Figure 5:**
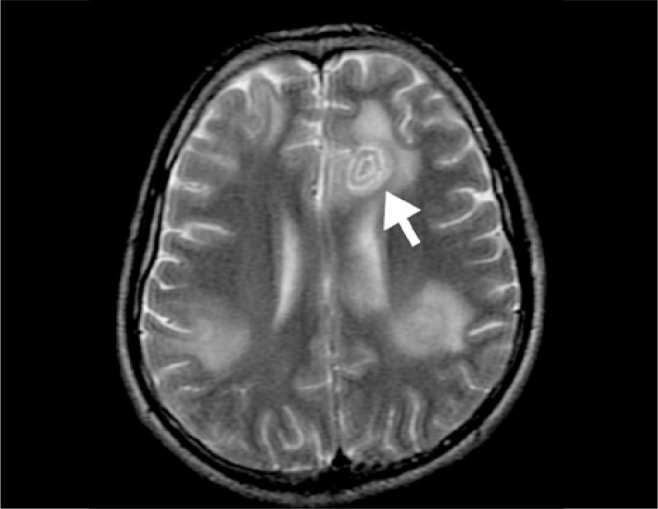
MRI of a 25-year-old male patient with AIDS, after an ini-tial seizure episode. T2WI showing a lesion with an apparent con-centric target sign, located in the left cingulate gyrus (arrow). Other focal lesions have a core with hypointensity on T2WI.

Two specific findings on MRI are the eccentric tar-get sign and the concentric target sign^**(^36^,^37^)**^. Present in approximately 30% of cases, the eccentric target sign is depicted on contrast-enhanced T1WI and consists of an enhanced inner eccentric core, a hypointense inter-mediate zone, and an enhanced peripheral border^**(^38^)**^, as illustrated in Figure 4, with 95% specificity and 25% sensitivity for the diagnosis of cerebral toxoplasmosis. It may indicate hemorrhage or coagulative necrosis of the tissue, and can be accompanied by external areas of edema and inflammatory cell infiltration. The concentric target sign is depicted on T2WI or FLAIR sequences and is characterized by alternating patterns of concentric zones of hypointensity and hyperintensity in deep parenchymal lesions (Figure 5). It is believed to be more specific for cerebral toxoplasmosis than is the eccentric target sign, although its specificity and histopathological correlation require further validation^**(^39^-^41^)**^.

**Figure 6:**
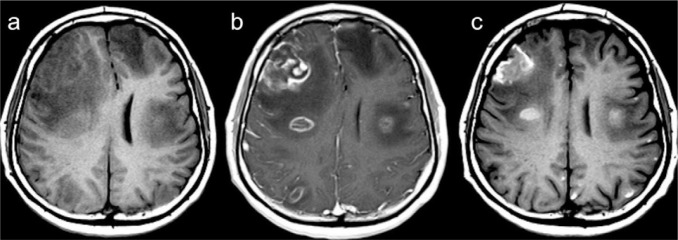
MRI scans of a patient with cerebral toxoplasmosis. Unenhanced axial T1WI (**A**) and contrast-enhanced axial T1WI (**B**) showing multiple intra-axial nodular lesions with ring enhancement (the right frontal lesion with concentric enhancement) and perilesional edema. In **C**, a follow-up scan showing hyperintensity of the lesions, which corresponds to subacute hemorrhage due to the specific treatment. Note also the reduction in the degree of vasogenic edema.

**Figure 7:**
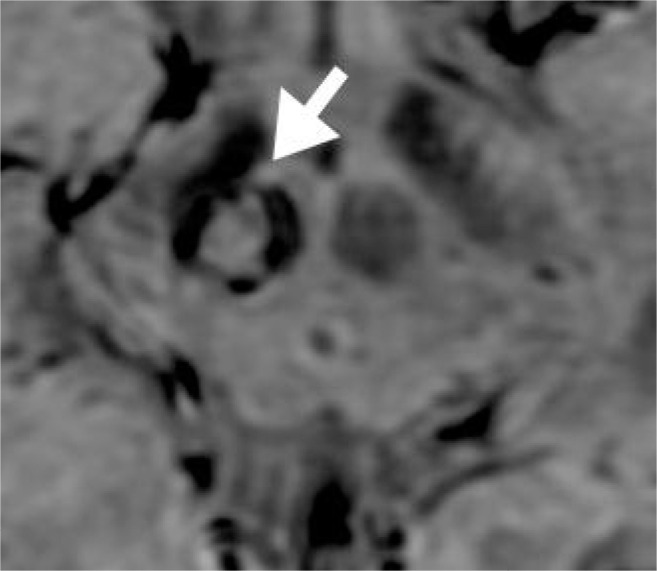
MRI scan of a patient with cerebral toxoplasmosis. Axial SWI showing a right midbrain lesion, with peripheral hypointensity (representing hemorrhage).

In the vast majority of patients with cerebral toxo-plasmosis, SWI shows foci of intralesional susceptibility signals, most probably representing hemorrhage, as il-lustrated in Figure 6 (on T1WI) and Figure 7 (on SWI). It is important to note how these findings aren’t unique to toxoplasmosis, although SWI techniques have superior sensitivity in detecting hemorrhagic lesions^**(^30^)**^. SWI im-ages are important to depict calcifications and hemorrhage as areas of hypointense signal intensity^**(^41^)**^.

### Advanced imaging techniques

Advanced imaging techniques are other important tools for identifying cerebral toxoplasmosis lesions. On DWI, restricted diffusion may be seen at the periphery of the lesions. However, the apparent diffusion coefficient (ADC) value is less reliable, with conflicting findings in the literature, in which there is a wide spectrum of ADC values, with varying results even between lesions in the same patient in some studies,^**(^33^)**^ whereas other studies have shown high ADC values (indicating that there is no restricted diffusion) for toxoplasmosis-related lesions and relatively lower ADC values for lymphoma-related lesions^**(^21^,^42^,^43^)**^.

Dynamic susceptibility contrast-enhanced T2-weight-ed gradient echo echo planar sequences acquired during the first pass of a standard dose of gadolinium-based contrast agent shows reduced relative cerebral blood volume (rCBV) in toxoplasmosis lesions and in the surrounding edema, which results from a lack of vasculature in the le-sions and vasoconstriction caused by increased interstitial pressure in the edema, respectively. The rCBV levels are lower in toxoplasmosis-related lesions than in lymphoma-related lesions, which facilitates the diagnosis^**(^44^)**^.

**Figure 8:**
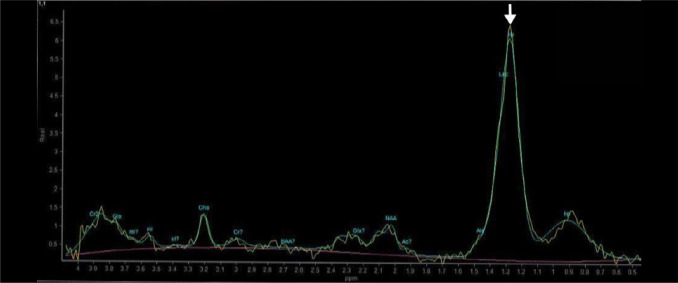
Single-voxel spectroscopy with short a echo time (31 ms) to investigate a right frontal lesion, showing a significant singlet peak at 1.3 ppm, indicating lactate/lipids (arrow), reflecting necrosis and anaerobic conditions.

Magnetic resonance spectroscopy provides informa-tion about the metabolic profile beyond the anatomical information and has been extensively used to evaluate brain focal lesions. In toxoplasmosis, there is classically the presence and elevation of lipid and lactate peaks, ac-companied by reduction of the other metabolites, such as creatine, choline, N-acetylaspartate, and myoinositol, as demonstrated in Figure 8^**(^45^)**^. However, the technique is only modestly capable of differentiating toxoplasmosis from lymphoma. The occurrence of an overlap in meta-bolic values between the two conditions is widely reported in the literature, although some studies also state the existence of unique profiles, such elevated choline levels in lymphomas^**(^34^)**^.

### Molecular imaging

Molecular imaging is increasingly employed, espe-cially with techniques such as SPECT and ^18^F-FDG PET. It has been shown that there is no uptake of thallium-201 by cerebral toxoplasmosis lesions on 201Tl SPECT. In addition, ^18^F-FDG PET/CT is an important diagnostic tool for cerebral toxoplasmosis, showing that there is less ^18^F-FDG uptake by toxoplasmosis lesions than by normal brain cortex.

**Figure 9:**
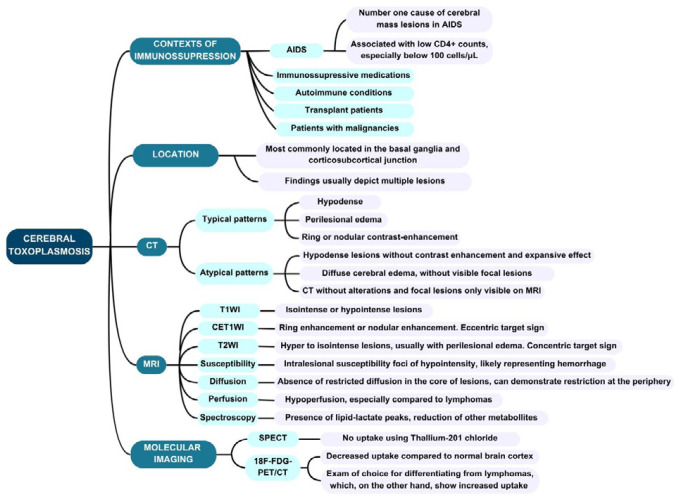
Schematic overview of cerebral toxoplasmosis. CE, contrast-enhanced.

The application of molecular imaging is useful in dif-ferentiating cerebral toxoplasmosis lesions from primary lymphomas, the latter characterized by higher uptake values. That being said, ^18^F-FDG-PET/CT should be implemented when neuroimaging findings are inconclu-sive or atypical for cerebral toxoplasmosis. In addition, the diagnostic value of 201Tl SPECT has decreased considerably due to increased uptake in patients with cerebral toxoplasmosis undergoing highly active anti-retroviral therapy^**(^46^)**^. Therefore, ^18^F-FDG-PET/CT is now the molecular imaging examination of choice, being particularly helpful in diagnosing patients suspected of having cerebral toxoplasmosis who do not respond to specific therapy. Figure 9 provides an overview of findings in cerebral toxoplasmosis.

### Specific clinical presentations

#### *T. gondii* myelitis

**Figure 10:**
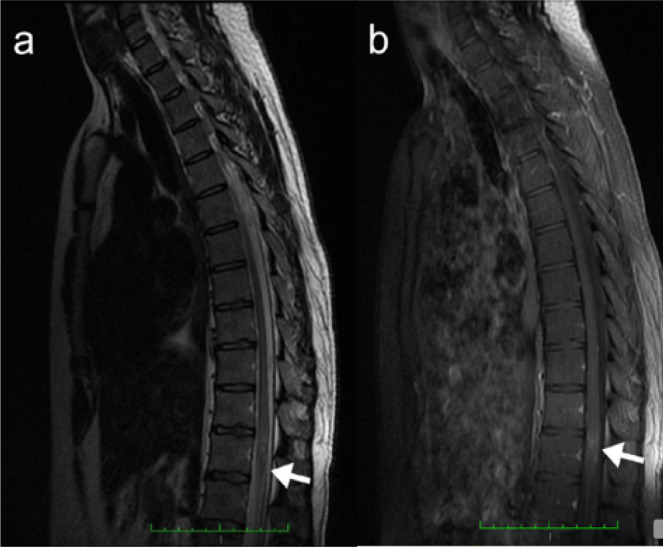
MRI scan of a patient with cerebral toxoplasmosis. Un-enhanced sagittal T2WI (**A**) and contrast-enhanced sagittal T1WI (**B**), showing an ill-defined poorly enhancing intramedullary lesion in the conus medullaris (arrows).

Cases of myelopathy associated with *T. gondii* infec-tion are rare, with few examples reported. Data in the literature suggest that myelitis is a finding present mostly in AIDS patients^**(^47^,^48^)**^. Classical symptoms related to spinal cord toxoplasmosis are motor loss (mainly affect-ing the lower extremities), paraparesis, bilateral sensory loss with specific spinal level, bladder dysfunction, and lumbar pain. Lastly, a review of the literature has shown that simultaneous cerebral and spinal cord involvement is common, as described in half of the analyzed cases^**(^49^)**^. In-fectious myelopathy is characterized by hyperintensity on T2WI sequences and hypointensity on T1WI sequences. In addition, the lesions are classically associated with lon-gitudinal spinal cord edema and intense, heterogeneous contrast enhancement. Toxoplasmosis-related lesions preferentially affect the thoracic spinal cord segments. Figure 10 shows a toxoplasmosis-related lesion in an unusual location (the conus medullaris).

### Cerebral toxoplasmosis and immune reconstitution inflammatory syndrome

Immune reconstitution inflammatory syndrome (IRIS) represents a paradoxical deterioration of an op-portunistic infection despite immunological recovery during treatment. In the case of cerebral toxoplasmosis, patients might develop a CNS-related form of IRIS, marking a worsening of the neurological condition. In patients with toxoplasmosis and HIV infection, there is an increase in the CD4+ cell count after antiretroviral therapy, which mediates the exacerbation via trafficking of CD4 and CD8 T lymphocytes to the nervous system.

The combination of cerebral toxoplasmosis and CNS-IRIS has rarely been described in the literature, with other opportunistic infections appearing as more likely differential diagnoses, such as chronic infection with a mycobacterial, viral, or fungal pathogen. Histopatho-logical proof of extensive inflammatory granulomatous processes does not affect the diagnosis or treatment of cerebral toxoplasmosis, only lengthening its natural clinical course^**(^50^-^52^)**^. In cases of CNS-IRIS caused by opportunistic pathogens, contrast-enhancing lesions in the brain are usually found. However, because cases of cerebral toxoplasmosis with and without IRIS have similar neuroimaging appearances, it is often necessary to take the clinical scenario and neuropathology into account in order to differentiate between them^**(^53^,^54^)**^.

### Differential diagnosis

Local epidemiology is an important factor to explore when determining the differential diagnosis of toxoplas-mosis. In high-income countries, primary CNS lymphoma (PCNSL) is the main differential diagnosis, whereas focal forms of cerebral tuberculosis (tuberculomas and tuberculous abscesses) are the main alternatives in low-and middle-income countries. Cerebral toxoplasmosis and PCNSL can both cause contrast-enhancing lesions with mass effect^**(^4^,^8^)**^.

### PCNSL

Patients with AIDS or other immunosuppressive conditions have a higher probability of PCNSL present-ing as multifocal lesions with necrosis and hemorrhage. Differentiating between PCNSL-related lesions and toxoplasmosis-related lesions may be difficult^**(^46^)**^.

#### Tuberculosis of the CNS

Tuberculomas and tuberculous abscesses usually ap-pear on CT images as solitary or multiple ring-enhancing lesions with a hypodense center and surrounding va-sogenic edema. A central calcification surrounded by a ring of enhancement, known as a target sign, is also common, although none of its appearances on CT are specific for tuberculomas. MRI is the preferred method for diagnosing possible tuberculomas is MRI, which reveals different possible patterns of enhancement: ir-regular, ring-like, open rings, and lobular. It should be borne in mind that tuberculomas can assume various patterns depending on the stage of the disease and the immunological status of the patient. As on CT scans, tuberculous abscesses appear as typical cerebral ab-scesses on MRI^**(^55^-^58^)**^, and restricted diffusion can be observed in the necrotic component. Leptomeningitis (with leptomeningeal enhancement) is also common in tuberculosis.

### Paracoccidioidomycosis

Paracoccidioidomycosis is a neglected tropical disease that is especially prevalent in Latin America, representing the leading cause of death due to systemic mycosis in im-munocompetent patients in Brazil^**(^59^-^61^)**^. Involvement of the CNS can manifest as intraparenchymal or meningeal lesions, with the latter being less common. The intraparen-chymal manifestations are granulomas classically located in the brain hemispheres, appearing as irregular lesions with mass effect, peripheral contrast-enhancement, and perilesional edema. The meningeal forms are associated with leptomeningeal contrast enhancement and small cortical nodules. Lastly, spectroscopy can show a treha-lose peak in paracoccidioidomycosis and other fungal infections**^(^62^-^65^)^**.

### Cryptococcosis

Cryptococcosis is an infectious disease caused by pathogenic encapsulated yeasts of the genus *Cryptococcus*, mainly *C. neoformans* and *C. gattii*. Like toxoplasmosis, cryptococcosis is especially harmful in immunocompro-mised hosts with AIDS. Typical imaging findings of cryp-tococcal meningoencephalitis include dilated perivascular spaces, pseudocysts, cryptococcomas, leptomeningeal or parenchymal enhancing lesions, and the hazy brain base sign.

Dilated perivascular spaces are common in immu-nocompetent and immunocompromised hosts alike^**(^66^)**^. The perivascular spaces are part of the glymphatic system, surrounding the vessels to protect them from mechani-cal injury and to allow chemical exchanges, mainly the depuration of toxins.

Cryptococcomas are intra-axial cryptococcal granulo-mas that are more common in immunocompetent hosts. These lesions usually show hypointensity on T1WI and hyperintensity on T2WI, with a ring or nodular enhance-ment and surrounding vasogenic edema^**(^67^-^69^)**^.

Leptomeningeal involvement, associated with focal parenchymal edema, tends to be mild, similar to im-mune reactions. The most sensitive imaging modality to demonstrate these findings is a contrast-enhanced FLAIR sequence, which typically reveals infratentorial lesions. The hazy brain base sign represents a pattern seen in typical cryptococcal meningitis, probably indicating fungal yeast penetration of the basal parenchyma along the perivascular spaces^**(^70^)**^.

### Future directions

The diagnosis of cerebral toxoplasmosis remains a challenging task. Although radiological findings can reli-ably help the process, the diagnosis is confirmed only if the patient responds to the specific treatment, because the symptoms and signs overlap with those of the dif-ferential diagnoses. Therefore, it is imperative to further evaluate new diagnostic modalities, such as the use of biomarkers that could help diagnose and possibly assess the treatment response. Molecular biology methods have great diagnostic potential, substantiating the need for a cost-effective technique that is highly sensitive and specific. In addition, identifying genetic risk factors for reactivation of cerebral toxoplasmosis should facilitate the stratification of patients. Furthermore, the study of imaging techniques already in use should continue, to better evaluate their sensitivity and specificity in diagnos-ing cerebral toxoplasmosis.

## CONCLUSION

Cerebral toxoplasmosis is a relevant condition in im-munosuppressed and congenitally infected individuals, being the leading cause of cerebral mass lesions in patients with AIDS. Encephalitis represents the most common clinical manifestation, typically with multiple lesions in the basal ganglia. However, it is a multifaceted condition presenting in various patterns, including the rare occur-rence of infectious myelitis. Advanced MRI techniques may aid in the diagnosis, because typical toxoplasmosis-related lesions do not demonstrate restricted diffusion in their core, do not have increased perfusion (i.e., show no increase in rCBV values), and, on spectroscopy, may show an increase in the peaks of lipids and lactate, with reductions in the other metabolites.

After reviewing the literature, we can state that neu-roimaging, especially MRI, is an essential tool for the early diagnosis of toxoplasmosis, for the evaluation of its initial extent, and for monitoring treatment responses. We found that CT and MRI consistently demonstrate findings characteristic of the disease, with advanced techniques and nuclear medicine studies improving the distinction from differential diagnoses, most importantly, PCNSL. Most patients respond favorably to treatment, although residual complications such as calcifications and intralesional hemorrhage have been reported. The characteristic eccentric target sign was reported in only a small portion of the studies included in our review, with no mention of the concentric target sign. Therefore, a multimodal imaging approach is essential to increase diagnostic accuracy and support clinical decision-making.
